# Expanding the Limits of Structural Characterization of Marine Dissolved Organic Matter Using Nonuniform Sampling Frequency-Reversed Edited HSQC NMR

**DOI:** 10.1021/acs.analchem.3c02923

**Published:** 2023-09-19

**Authors:** Sahithya Phani Babu Vemulapalli, Christian Griesinger, Thorsten Dittmar

**Affiliations:** † Research Group for Marine Geochemistry, Institute for Chemistry and Biology of the Marine Environment (ICBM), University of Oldenburg, 26129 Oldenburg, Germany; ‡ Department of NMR Based Structural Biology, Max Planck Institute (MPI) for Multidisciplinary Sciences, 37077 Göttingen, Germany; § Helmholtz Institute for Functional Marine Biodiversity at the University of Oldenburg (HIFMB), 26129 Oldenburg, Germany

## Abstract

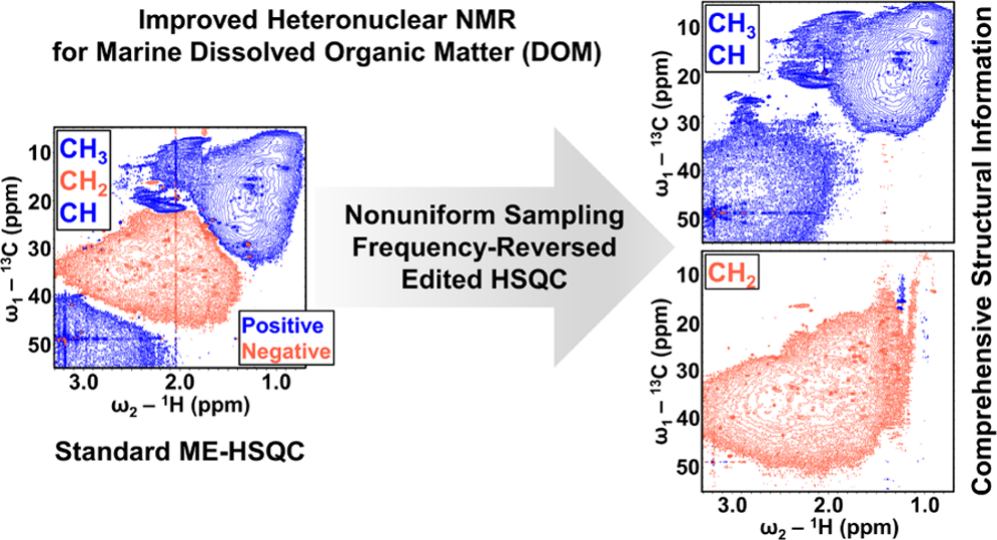

The multiplicity-edited heteronuclear single quantum correlation (ME-HSQC) NMR method is widely used for the structural characterization of marine dissolved organic matter (DOM), which is a complex molecular mixture comprising millions of individual compounds. However, the standard ME-HSQC suffers from significant signal cancellation and subsequent loss of crucial structural information due to the overlap between CH_3_/CH (positive) and CH_2_ (negative) cross-peaks in overcrowded regions. This study introduces nonuniform sampling in frequency-reversed ME-HSQC (NUS FR-ME-HSQC), highlighting its remarkable potential for the comprehensive structural characterization of marine DOM. By reversing the frequency of CH_2_ cross-peaks into an empty region, the FR-ME-HSQC method effectively simplifies the spectra and eliminates signal cancellation. We demonstrate that nonuniform sampling enables the acquisition of comparable spectra in half the time or significantly enhances the sensitivity in time-equivalent spectra. Comparative analysis also identifies vulnerable CH_2_ cross-peaks in the standard ME-HSQC that coincide with CH_3_ and CH cross-peaks, resulting in the loss of critical structural details. In contrast, the NUS FR-ME-HSQC retains these missing correlations, enabling in-depth characterization of marine DOM. These findings highlight the potential of NUS FR-ME-HSQC as an advanced NMR technique that effectively addresses challenges such as signal overcrowding and prolonged experimental times, enabling the thorough investigation of complex mixtures with implications in several fields, including chemistry, metabolomics, and environmental sciences. The advantages of NUS FR-ME-HSQC are experimentally demonstrated on two solid-phase-extracted DOM (SPE-DOM) samples from the surface and deep ocean. With this new technology, differences in the composition of DOM from various aquatic environments can be assigned to individual molecules.

## Introduction

Marine dissolved organic matter (DOM) is an important component of the global and marine geochemical cycles, which has accumulated (∼662 Pg C) over millennia to form one of the largest active carbon reservoirs on Earth’s surface.^1^ Uncovering the structural composition and cycling of marine DOM is crucial in understanding its biogeochemistry.^2^ The molecular diversity of marine DOM across oceanic provinces has been extensively investigated on a molecular formula level via ultrahigh-resolution Fourier transform ion cyclotron resonance mass spectrometry (FT-ICR-MS) analyses.^3−7^ However, the structural composition of marine DOM on the molecular level remains poorly characterized.^8^

In this regard, high-field nuclear magnetic resonance (NMR) spectroscopy serves as an invaluable tool for the in-depth structural analysis of marine DOM.^3,9−11^ Particularly, the multiplicity-edited heteronuclear single quantum correlation (ME-HSQC)^12−15^ technique is widely employed for the qualitative and semiquantitative analysis of dissolved organic matter.^3,16−20^ However, the analysis of highly complex mixtures, such as marine DOM, poses challenges to conventional analytical techniques due to the presence of millions of individual compounds at very dilute concentrations.^6^ The standard ME-HSQC method, while effective for individual isolated compounds and simpler mixtures, falls short when applied to an extremely complicated marine DOM. A major drawback of the standard ME-HSQC technique is the signal cancellation arising from the overlap of negative CH_2_ cross-peaks with those of positive CH_3_ and CH cross-peaks when recorded at low digital resolution in the indirect dimension (ω_1_) of the two-dimensional (2D) NMR experiment.^21^ This hampers the accurate interpretation of carbon–proton correlations, leading to the loss of valuable structural information. To avoid the overlap of cross-peaks in the standard ME-HSQC, one must increase the digital resolution by recording a large number of *t*_1_ increments in the indirect dimension (ω_1_). This approach is impractical as the experiment time increases proportionally with *t*_1_ increments, and even a single ME-HSQC requires more than a week of instrument time,^16^ particularly for mass-limited DOM samples from deep and remote locations. Most researchers do not have access to this amount of high-field NMR measurement time. Nevertheless, the loss of structural information is unavoidable even in the ME-HSQC recorded with high digital resolution, where the positions of CH_3_/CH and CH_2_ cross-peaks are similar.

It is crucial to mention here that there is no single marine DOM extraction method that yields 100% efficiency.^22−24^ Even the widely used solid-phase extraction (SPE)^22,24^ method yields approximately 61% SPE-DOM. Consequently, most of the studies focused on the structural characterization of marine DOM have been carried out on an “operationally defined fraction” rather than the “entirety” of DOM. Additionally, the loss of structural information due to the shortcomings of conventional techniques necessitates the need for advanced NMR methods capable of providing comprehensive structural information on marine DOM. In this context, this study presents the first report on the application of the frequency-reversed ME-HSQC (FR-ME-HSQC)^21^ method, combined with nonuniform sampling (NUS),^25−27^ as a promising approach for expanding the limits of structural characterization of marine DOM. By reversing the frequency of CH_2_ cross-peaks, FR-ME-HSQC simplifies overcrowded regions and eliminates signal cancellation, even at low digital resolution. The FR-ME-HSQC offers a potential solution to retaining important structural details and substantially improving the analysis of highly complex mixtures. Additionally, the implementation of 50% nonuniform sampling (i) reduces the measurement time by half without compromising spectral quality or (ii) significantly enhances the signal intensity for the same total measurement time, compared to conventional uniform sampling for marine DOM as demonstrated for two-dimensional heteronuclear single quantum correlation (2D HSQC)^28^ spectroscopy and two-dimensional correlation spectroscopy (2D COSY).^29^ Signal envelope-matched nonuniform sampling can enhance sensitivity by capturing the majority of the signal while discarding the noise. Farooq et al.^28^ demonstrated that *T*_2_-weighted exponential sampling offers improved sensitivity compared to non-*T*_2_-weighted nonuniform sampling for the natural organic matter. Similarly, sinusoidal-weighted Poisson-gap sampling concentrates most samples at the beginning of the time-domain data,^30^ where the majority of the signal is expected. This approach has been shown to be advantageous for exponentially decaying time-domain data, as is the case with HSQC. For the 2D NMR spectra that exhibit clustered sparsity, the combination of Poisson-gap sampling^30,31^ and compressed sensing (CS)^32^ reconstruction has been reported to perform well compared to other approaches.^33^ The HSQC spectra of marine DOM also demonstrate this clustered sparsity, making the sinusoidal-weighted Poisson-gap sampling and compressed sensing reconstruction using the iterative soft thresholding (IST)^34^ algorithm the preferred choice employed throughout this study. The findings highlight the efficacy of NUS FR-ME-HSQC in overcoming the challenges associated with complex mixtures, such as marine DOM and pave the way for a more comprehensive understanding of their molecular composition.

It is worth mentioning here that the multiplicity-separated HSQC (MS-HSQC) technique, as described by Chen et al.^35^ provides two separate spectra for CH_2_ and CH/CH_3_ structural groups. Similar to the in-phase-anti-phase (IPAP) HSQC approach, MS-HSQC yields two distinct spectra for CH_2_ and CH/CH_3_ groups by directly adding and subtracting the interleaved ^1^*J*_XH_-active and ^1^*J*_XH_-inactive HSQC spectra. Exploring the performance of the multiplicity-separated HSQC technique in resolving complex natural organic mixtures is interesting; nevertheless, it falls beyond the scope of this manuscript.

The structural characterization of highly complex mixtures, such as dissolved organic matter (DOM), is an evolving, yet challenging field. The potential of advanced NMR spectroscopic techniques has not been explored to its fullest. The nonuniform sampling approach is very powerful in this context.

Overall, this article serves as a critical step toward introducing advanced analytical tools that enable the thorough investigation of complex molecular mixtures, with implications in various fields, including marine and aquatic sciences, chemistry, and metabolomics.

## Experimental Section

### Marine SPE-DOM

Two DOM samples (Natural Energy Laboratory of Hawaii Authority) from the surface (21 m sampling depth) and deep ocean (674 m sampling depth) were used in the current study.^22^ The latter is the North Equatorial Pacific Intermediate Water (NEqPIW). The NMR sample preparation of both the surface and deep ocean SPE-DOM is described elsewhere.^29^ A 100 mg sample of SPE-DOM was dissolved in 200 μL of 99.95% CD_3_OD solvent, and the solution was transferred to the 3 mm NMR tubes.

### NMR Spectroscopy

NMR spectra were acquired on Bruker Avance III HD 900 MHz (for ^1^H) and Avance NEO 800 MHz (for ^1^H) instruments equipped with 5 and 3 mm TCI cryoprobes, respectively. The standard ME-HSQC spectra were recorded using the pulse sequence hsqcedetgpsisp2.4. The frequency-reversed ME-HSQC was recorded using the pulse sequence hsqcedgpphsp_rev.2. Sinusoidal-weighted Poisson-gap sampling schedules were generated using the Schedule Generator Version 3.0 provided on nus@HMS webpage (http://gwagner.med.harvard.edu/intranet/hmsIST/gensched_new.html). The time-domain data points were set to 3072 in the F2 dimension and 1024 in the F1 dimension. The spectral width was set at 10.8 kHz for F2 and 34.5 kHz for F1, effectively covering a wide range of frequencies. Prior to data collection, 128 dummy scans were performed, followed by 32 scans for actual measurement. A higher number of dummy scans is employed to counteract the sample heating caused by the carbon broadband decoupling element and to allow the sample to reach its thermal equilibrium and steady state before acquiring the time-domain data. A relaxation delay of 2 s was implemented to ensure proper relaxation of the nuclear spins. Acquisition times of 0.142 s for F2 and 0.015 s for F1, with a fid resolution of 7 Hz for F2 and 67 Hz for F1, were used. The one-bond C–H coupling constant was set to 145 Hz. For a complete description of the acquisition and processing parameters, refer to Tables S1 and S2. All experiments were performed at 298 K. All of the spectra were processed with Topspin 4.2.0 (Bruker BioSpin, Germany) and visualized using Sparky.^36^

## Results and Discussion

### Frequency-Reversed ME-HSQC for the Marine DOM

**Figure 1:**
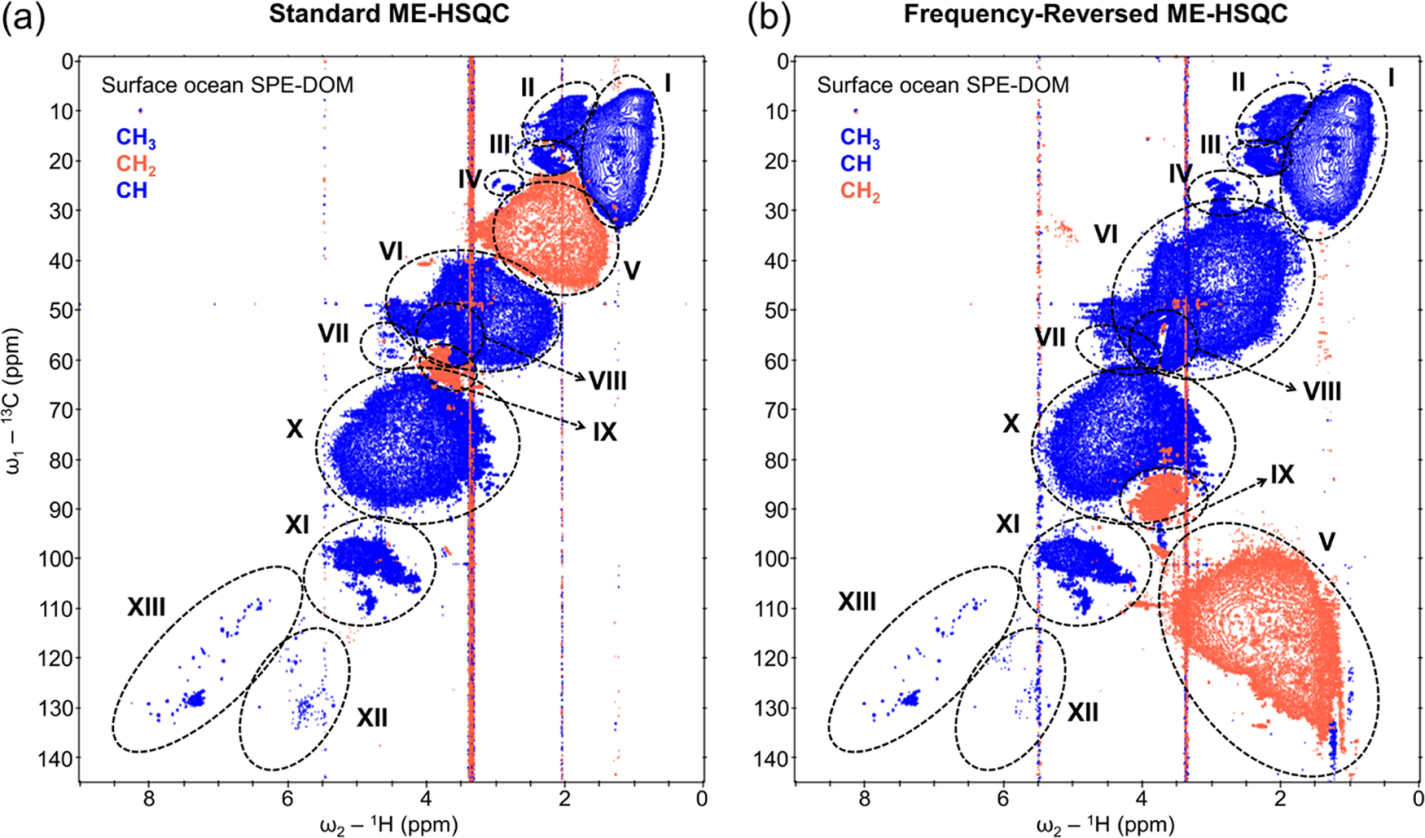
NMR spectra of surface ocean SPE-DOM. Comparison of the standard ME-HSQC (a) and FR-ME-HSQC (b) of surface ocean SPE-DOM in CD_3_OD solvent, recorded on a 900 MHz (for ^1^H) NMR instrument. The blue (positive) cross-peaks represent the CH_3_ and CH correlations, and the red (negative) cross-peaks represent the CH_2_ correlations. Key structural assignments are as follows: **I**, diverse aliphatic groups and terminal methyl groups; **II**, *N*-acetyl/*O*-acetyl/S–**CH**_**3**_ and C=C–**CH**_**3**_; **III**, acetates (−OOC–**CH**_**3**_) and aromatic methyl groups (Ar–**CH**_**3**_); **IV**, *N*-methyl groups (–HN–**CH**_**3**_); **V**, diverse **CH**_**2**_ groups and carboxyl-rich alicyclic molecules (CRAM); **VI**, diverse **CH** groups and CRAM; **VII**, α groups (**C**_**α**_**H**_**α**_) in biomolecules; **VIII**, methyl esters (−OCO–**CH**_**3**_) and methoxy groups (–O–**CH**_**3**_); **IX**, **CH**_**2**_ in carbohydrates, and bonded to oxygen (–O–**CH**_**2**_–); **X**, diverse **CH** groups, mainly from carbohydrates; **XI**, anomeric **CH** in carbohydrates; **XII**, olefinic **CH**; **XIII**, **CH** groups in aromatic, heterocyclic and polycyclic aromatic hydrocarbons (PAHs). Structural group assignments are modified based on ref.^16−18,37−40^

Marine DOM is a complex mixture of hundreds of thousands of individual compounds, each existing at very dilute concentrations.^6^ Despite being recorded at the high-field 800 and 900 MHz NMR instruments, the standard ME-HSQC spectra of surface and deep ocean SPE-DOM (Figures 1a and S1a, respectively) exhibit broad and unresolved cross-peak patterns, reflecting the remarkable molecular complexity. Analyzing the ME-HSQC of marine DOM on an atomic level is impossible; therefore, the spectral regions are categorized into key structural classes (Figures 1 and S1). Despite its ability to discriminate between CH_3_/CH and CH_2_ correlations, the standard ME-HSQC suffers from significant signal loss, thereby compromising structural information (Figures 1a and S1a). In contrast, FR-ME-HSQC (Figures 1b and S1b) astonishingly simplifies the overcrowded regions by reversing the frequency of CH_2_ correlations into the signal-free spectral region, without the need for further increase in the digital resolution in the indirect dimension (ω_1_).

**Figure 2:**
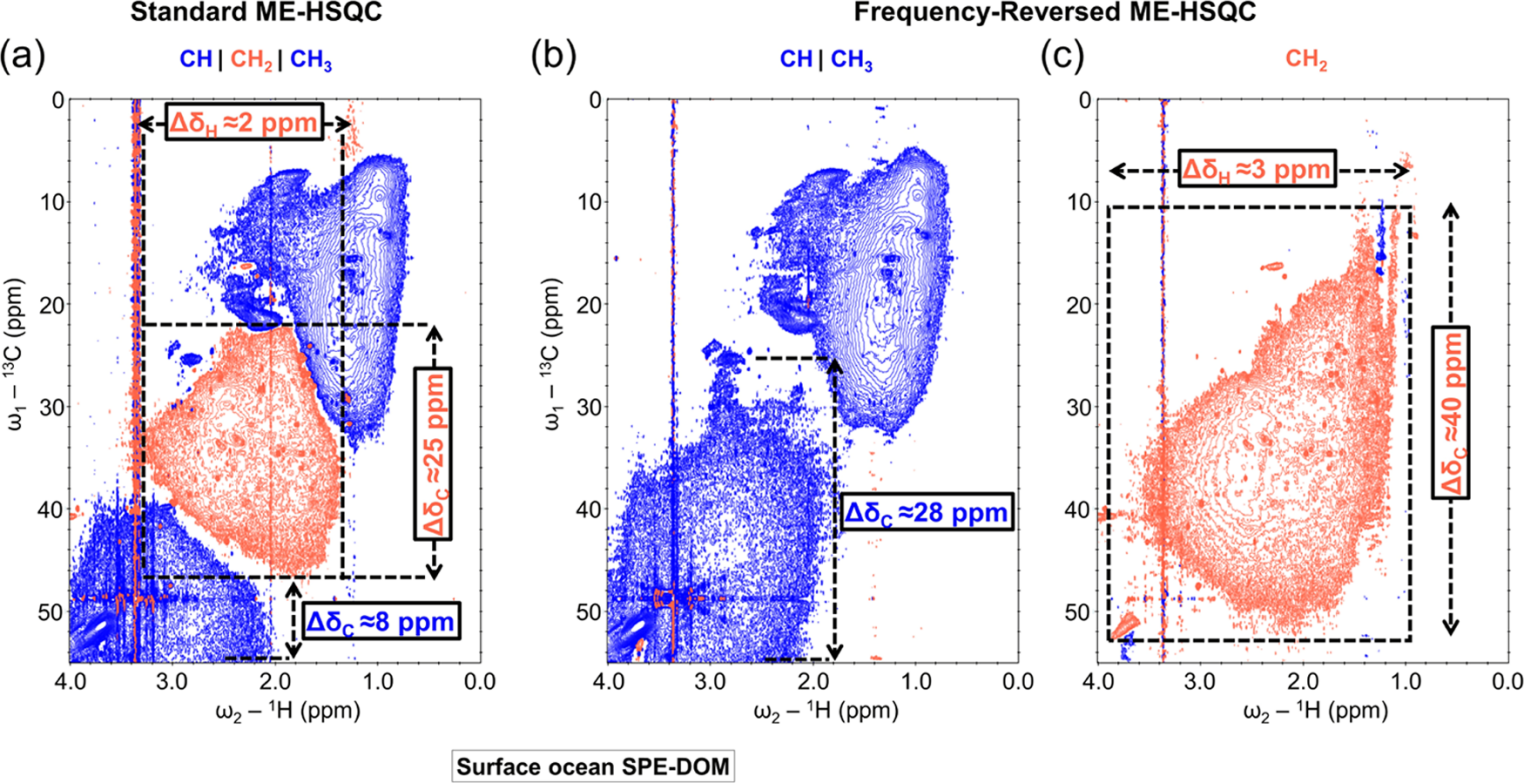
NMR spectra of surface ocean SPE-DOM. Comparison of a selected region of standard ME-HSQC (a), FR-ME-HSQC with CH and CH_3_ correlations (b), and FR-ME-HSQC with CH_2_ correlations (c) of surface ocean SPE-DOM in CD_3_OD solvent, recorded on a 900 MHz (for ^1^H) NMR instrument. The blue (positive amplitude) cross-peaks represent the CH_3_ and CH correlations, and the red (negative amplitude) cross-peaks represent the CH_2_ correlations. The spectrum in (c) is inverted in the indirect dimension (ω_1_) during the Fourier transformation to display the original frequency of the CH_2_ correlations. Dotted lines indicate the ranges of ^13^C and ^1^H chemical shifts for different carbon–proton correlations.

The significant increase in the volume of the blue and red lobes in the FR-ME-HSQC (Figure 2b,c) compared to the standard ME-HSQC (Figure 2a) of surface SPE-DOM clearly indicates an enhanced structural information content. The carbon–proton correlations of CH_2_ groups in marine DOM were observed only in a limited region of standard ME-HSQC, primarily due to signal cancellation in the crowded region. Carbon chemical shifts spanned approximately 25 ppm, while proton chemical shifts covered about 2 ppm (Figure 2a). In contrast, the FR-ME-HSQC effectively retained the diverse structural information on CH_2_ groups, as indicated by the expanded range of carbon chemical shifts (approx. 40 ppm) and proton chemical shifts (around 3 ppm) (Figure 2c). Furthermore, in a specific region of the standard ME-HSQC, the carbon chemical shifts of CH correlations were confined to a smaller range (approx. 8 ppm) (Figure 2a), whereas the FR-ME-HSQC exhibited an increased carbon chemical shift range for these CH cross-peaks, spanning approx. 28 ppm (Figure 2b). This clear enhancement highlights the potential of FR-ME-HSQC in providing comprehensive structural information on marine DOM. A substantial increase in the peak capacity (structural features) of deep ocean SPE-DOM is evident from the comparison of selected regions of the standard ME-HSQC (Figure S2a) and FR-ME-HSQC (Figure S2b,c).

In the comparison of another distinct region, where the CH_2_ cross-peaks of oxygenated methylene groups primarily derived from carbohydrates overlapped with the C_α_H_α_ correlations of biomolecules, a clear loss of signal is observed in the standard ME-HSQC spectra of the surface and deep ocean SPE-DOM (Figure S3a,d, respectively). However, FR-ME-HSQC (Figure S3b,c,e,f) successfully retained the missing carbon–proton correlations. Notably, FR-ME-HSQC exhibited an increased number of carbon–proton cross-peaks for CH (Figure S3b,e), as well as CH_2_ (Figure S3c,f) groups.

The FR-ME-HSQC spectra of surface and deep ocean SPE-DOM exhibit shared signals, but notable and distinct sharp signals are observed to be different (Figure S3). In the FR-ME-HSQC of deep ocean SPE-DOM, the carbon–proton correlation at 62.7–3.83 ppm is clearly visible (Figure S3e), while it is absent in standard ME-HSQC (Figure S3d). Conversely, this correlation is not observed in the surface ocean SPE-DOM. Additionally, the carbon–proton cross-peaks observed at 61.2–4.20, 61.2–3.83, 62.6–3.79, and 63.2–3.79 ppm in the FR-ME-HSQC (Figure S3b) of the surface ocean SPE-DOM are lost in the standard ME-HSQC (Figure S3a). These newly obtained carbon–proton correlations may correspond to the isoleucine (61.6–4.16 ppm), valine (63.0–3.60 ppm), and threonine (63.2–3.57 ppm) C_α_H_α_ resonances (Table S5), as verified using data from Biological Magnetic Resonance Data Bank (BMRB).^41^ Notably, these resonances are not present in the deep ocean SPE-DOM. The FR-ME-HSQC revealed profound differences in the molecular composition between surface and deep ocean SPE-DOM, which otherwise appear structurally more similar when analyzed using the standard ME-HSQC. These findings highlight the FR-ME-HSQC technique’s ability to reveal the molecular diversity between DOM from various freshwater and marine ecosystems. A more comprehensive analysis of these differences will be conducted in future studies, which are outside the scope of this manuscript.

### Nonuniform Sampling Frequency-Reversed ME-HSQC for the Marine DOM

**Figure 3:**
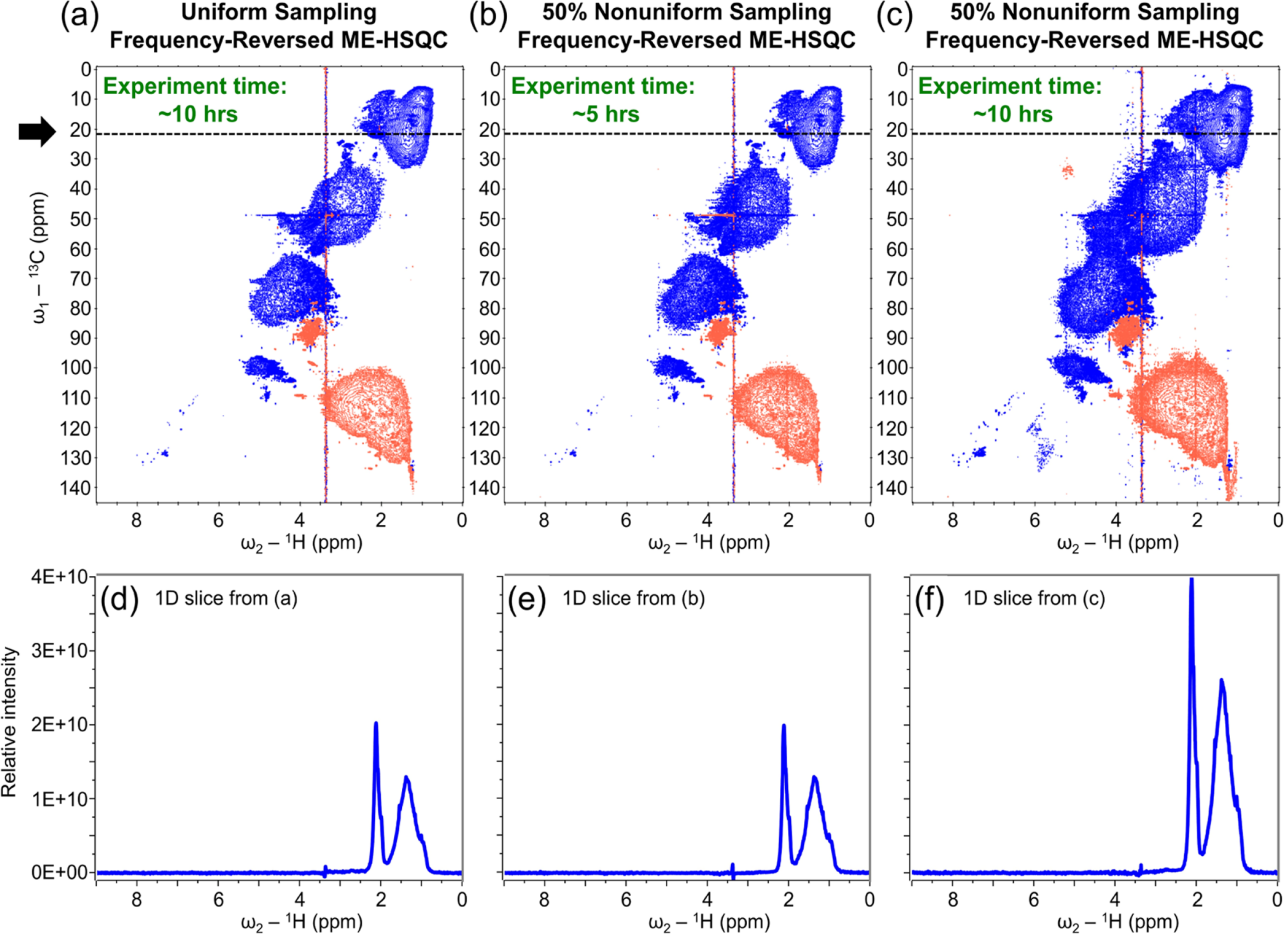
NMR spectra of surface ocean SPE-DOM. Comparison of the conventional uniformly sampled FR-ME-HSQC (a), 50% NUS FR-ME-HSQC recorded in half the time (b), and time-equivalent 50% NUS FR-ME-HSQC (c) of surface ocean SPE-DOM in CD_3_OD solvent, recorded on an 800 MHz (for ^1^H) NMR instrument. The 1D slices (d–f) are extracted along the direct dimension (ω_2_) of (a–c), respectively, at a position indicated by the dotted lines and an arrow.

The conventional uniformly sampled FR-ME-HSQC of 100 mg each of surface and deep ocean SPE-DOM, recorded on the high-field 800 MHz NMR instrument, required approx. 10 h of experimental time (Figures 3a and S4a, respectively). For mass-limited (≈1 mg) marine DOM, the FR-ME-HSQC may even require longer measurement times assuming it is recorded with a higher number of scans to obtain a reasonably high signal-to-noise ratio. Consequently, there is a need for advanced NMR approaches to speed up the acquisition of 2D NMR experiments. To circumvent this issue, we demonstrate the advantages of nonuniform sampling (NUS) in 2D FR-ME-HSQC for marine DOM.

Implementing 50% NUS in a 2D FR-ME-HSQC allowed for a 2-fold reduction in measurement time (Figures 3b and S4b) without sacrificing spectral quality. The NUS FR-ME-HSQC recorded in half the time is essentially indistinguishable from the conventional uniformly sampled FR-ME-HSQC of surface (Figure 3d,e) and deep (Figure S4d,e) ocean SPE-DOM, as seen from the comparison of 1D slices extracted from the 2D NMR spectra. Time-equivalent NUS FR-ME-HSQC of surface and deep ocean SPE-DOM, recorded with twice the number of scans compared with the conventional uniformly sampled counterpart, showed significantly enhanced detection sensitivity (Figures 3c and S4c), thereby enabling efficient structural analysis of mass-limited complex molecular mixtures. As expected, the relative intensity of the signals in the time-equivalent NUS FR-ME-HSQC is increased by a factor of ≈2 compared to the conventional FR-ME-HSQC of surface (Figure 3d,3f) and deep (Figure S4d,f) ocean SPE-DOM that are both not mass-limited here. The key benefit of the enhanced sensitivity in the nonuniformly sampled FR-ME-HSQC is its significant improvement in detecting very weak signals that are nearly obscured within the noise of the standard uniformly sampled FR-ME-HSQC. In this context, the term “sensitivity” refers to “detection sensitivity”, as described by Hyberts et al.,^42^ which denotes the probability to detect weak peaks. The detection sensitivity of the time-equivalent nonuniformly sampled FR-ME-HSQC spectra increases due to the higher number of scans, thereby enhancing the information content of the multidimensional NMR spectra of natural organic matter. It is important to mention here that the standard signal-to-noise ratio (SNR) cannot be directly used as a sensitivity indicator for NUS due to the nonlinearity of the reconstruction process.^42,43^

The original frequency of the reversed CH_2_ cross-peaks in FR-ME-HSQC can be obtained as follows:
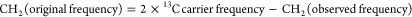
Consequently, the observed frequency of reversed CH_2_ signals depends on both the spectral width and the carrier frequency of the indirect dimension (ω_1_). By selecting suitable experimental parameters, it is straightforward to separate both of the CH_2_ regions without encountering any interference with the CH and CH_3_ correlations (Figure S5), at some cost of experimental time.

To identify the vulnerable CH_3_ and CH cross-peaks that could overlap with the CH_2_ cross-peaks in the conventional ME-HSQC, we compared the ME-HSQC spectra of marine DOM with the carbon–proton chemical shifts obtained from BMRB. The carbon–proton correlations of methoxy (–O–**CH**_**3**_), acetyl (−CO–**CH**_**3**_), *N*-methyl (−NH–**CH**_**3**_), and olefinic methyl (C=(COOH)C–**CH**_**3**_) groups (Table S3) are found to fall into the CH_2_ region, resulting in the loss of important structural information on compounds containing the aforementioned methyl groups in marine DOM. Numerous CH_2_ groups that are susceptible to signal cancellation in the regular ME-HSQC are identified (Table S4): **CH**_**2**_ groups of CRAM, long-chain fatty acids, monoglycerides, phospholipids, and aliphatic molecules; **CH**_**2**_ bound to aromatic, acid, amine, and guanidine groups; β-**CH**_**2**_ of amino acids; ring-**CH**_**2**_ of carbohydrates; and some of the **CH**_**2**_ groups of steroids, to mention a few. The structural information on compounds containing terminal methine groups (–(CH_3_)_2_**CH**) and **C**_**α**_**H**_**α**_ groups of amino acids (Table S5) and biomolecules is expected to be missing in the standard ME-HSQC. In contrast, the NUS FR-ME-HSQC has been proven to retain all of the aforementioned missing carbon–proton correlations, thereby facilitating the in-depth structural characterization of marine DOM.

## Conclusions

In summary, our study demonstrates the potential of NUS FR-ME-HSQC as an advanced NMR method for the comprehensive structural characterization of marine DOM. The limitations associated with the standard ME-HSQC technique, such as signal cancellation and loss of structural information due to overcrowded spectral regions, are overcome through the frequency-reversed approach. By reversing the frequency of CH_2_ cross-peaks to signal-free regions, FR-ME-HSQC significantly enhances the structural information content. The implementation of nonuniform sampling in FR-ME-HSQC allows the acquisition of spectra comparable to the conventional uniformly sampled counterpart in half of the time or significantly enhances the signal intensity for the same total measurement time. The significantly increased carbon–proton correlations in NUS FR-ME-HSQC spectra highlight its improved characterization capabilities compared to the standard ME-HSQC. The identification of vulnerable CH_2_ cross-peaks in the conventional ME-HSQC that overlap with CH_3_ and CH cross-peaks signifies the loss of essential structural information pertaining to amino acids; biomolecules; CRAM; carbohydrates; methoxy, acetyl, *N*-methyl, and phosphorus-containing organic compounds; polyhydroxy compounds; steroids; unsaturated compounds; long-chain fatty acids; lipids; and aliphatic compounds of marine DOM. This crucial structural information is effectively retained in the NUS FR-ME-HSQC of marine DOM. The NUS FR-ME-HSQC enables semiquantitative analysis of marine DOM, providing insights into the relative abundance of key structural groups. Overall, the findings emphasize the necessity of advanced NMR techniques such as NUS FR-ME-HSQC for the comprehensive analysis of intricate molecular mixtures in general and marine DOM in particular. This method offers an elegant solution to the challenges posed by mass-limited marine DOM samples, overcrowded spectra, and longer measurement times, enabling researchers to gain valuable insights into the molecular diversity of marine DOM across different oceanic provinces.

## Data Availability

The NMR data that support the findings of this study was uploaded to Edmond (10.17617/3.ULTCWJ), an open research data repository of the Max Planck Society.

## Supporting Information Available

NMR acquisition and processing parameters for the marine DOM (Tables S1 and S2); 2D HSQC spectra of the deep ocean SPE-DOM (Figure S1); expanded regions of 2D HSQC spectra of the deep ocean SPE-DOM (Figure S2); expanded regions of 2D HSQC spectra of the surface and deep ocean SPE-DOM (Figure S3); nonuniform sampling 2D HSQC spectra of the deep ocean SPE-DOM (Figure S4); 2D HSQC spectra of the surface ocean SPE-DOM (Figure S5); ^13^C and ^1^H chemical shifts of CH_3_, CH_2_, and CH groups of the representative molecules (Tables S3, S4, and S5); Bruker pulse program for FR-ME-HSQC; and references (PDF)
