# Supplementary material for: Expanding the Limits of Structural Characterization of Marine Dissolved Organic Matter Using Nonuniform Sampling Frequency-Reversed Edited HSQC NMR
Source: Anal Chem. 2023 Sep 19;95(39):14770–6. doi: 10.1021/acs.analchem.3c02923 (PMC10551856; doi:10.1021/acs.analchem.3c02923)
Supplement: Supplementary file 1 — ac3c02923_si_001.pdf [file ac3c02923_si_001.pdf]

Supporting Information

Expanding the Limits of Structural Characterization of Marine Dissolved Organic Matter Using Nonuniform Sampling Frequency-Reversed Edited HSQC NMR

Sahithya Phani Babu Vemulapalli,[*, †] Christian Griesinger,[‡] and Thorsten Dittmar[*,†,§]

[†] Research Group for Marine Geochemistry, Institute for Chemistry and Biology of the Marine Environment (ICBM), University of Oldenburg, 26129 Oldenburg, Germany
[‡] Department of NMR Based Structural Biology, Max Planck Institute (MPI) for Multidisciplinary Sciences, 37077 Göttingen, Germany
[§] Helmholtz Institute for Functional Marine Biodiversity at the University of Oldenburg (HIFMB), 26129 Oldenburg, Germany

*Correspondence:

sahithya.phani.babu.vemulapalli@uni-oldenburg.de

thorsten.dittmar@uni-oldenburg.de

Table of contents

Table S1. The NMR acquisition and processing parameters at the 900 MHz……….………….3

Table S2. The NMR acquisition and processing parameters at the 800 MHz……….………….4

Figure S1. 2D HSQC spectra of the deep ocean SPE-DOM……………….………………5

Figure S2. Expanded regions of 2D HSQC spectra of the deep ocean SPE-DOM……....……6

Figure S3. Expanded regions of 2D HSQC spectra of the surface and deep ocean SPE-DOM.....7

Figure S4. Nonuniform sampling 2D HSQC spectra of the deep ocean SPE-DOM……………8

Figure S5. The effect of ^{13}C spectral width and carrier frequency on the separation of CH_2 regions in the FR-ME-HSQC………………………………………………………9

Table S3. ^{13}C and ^{1}H chemical shifts of "CH_3" groups of the representative molecules……….10

Table S4. ^{13}C and ^{1}H chemical shifts of "CH_2" groups of the representative molecules……...11

Table S5. ^{13}C and ^{1}H chemical shifts of "CH" groups of the representative molecules………14

The Bruker pulse program used for recording the FR-ME-HSQC spectra……………………15

References…………………………………………………………………………….20

Table S1. Acquisition and processing parameters for the marine SPE-DOM at the 900 MHz (for ^{1}H) NMR instrument.

**Acquisition parameters**	**Standard ME-HSQC**	**FR-ME-HSQC**
Pulse sequence	hsqcedetgpsisp2.4[1-6]	hsqcedgpphsp_rev.2[5, 7]
Acquisition mode (FnMODE)	Echo-Antiecho	States-TPPI
Time domain points (TD)	3072 (F2) & 1024 (F1)	3072 (F2) & 1024 (F1)
Dummy scans (DS)	128	128
Number of scans (NS)	32	32
Spectral width (SW), kHz	10.8 (F2) & 34.5 (F1)	10.8 (F2) & 34.5 (F1)
Acquisition time (AQ), sec	0.142 (F2) & 0.015 (F1)	0.142 (F2) & 0.015 (F1)
One-bond C-H coupling, $^1J_{CH}$, Hz	145	145
D1, sec	2	2
Options for zg	-	-DFLAG_INV
Trim pulse, µs	1000	-
180° shaped pulse for inversion	500 µs (Crp60,0.5,20.1)	500 µs (Crp60,0.5,20.1)
180° shaped pulse for refocusing	2000 µs (Crp60comp.4)	-
180° shaped pulse for adiabatic matched sweep‖	1413 µs (Crp60_xfilt.2)	1413 µs (Crp60_xfilt.2)
**Processing parameters**		
Zero filling (SI)	4096 (F2) & 2048 (F1)	4096 (F2) & 2048 (F1)
Window function (WDW)	QSINE; SSB 2 (F2) & QSINE; SSB 2 (F1)	QSINE; SSB 2 (F2) & QSINE; SSB 2 (F1)
Phasing mode (PH_mod)	pk (F2) & pk (F1)	pk (F2) & pk (F1)
Reverse spectrum during transform#	False (F2) & False (F1)	False (F2) & False (F1)
MC2 (FnMODE)	Echo-Antiecho	States-TPPI

‖180° shaped pulse for adiabatic matched sweep: 1730 µs at 600.13 MHz, otherwise: 1730*sqrt(600/x). Where x is the magnetic field employed (900 MHz for the experiments on marine DOM).

#For the spectra in Figures 2c, S2c, S3c, and S3f:
Reverse spectrum during transform: False (F2) & True (F1).

Table S2. Acquisition and processing parameters for the marine SPE-DOM at the 800 MHz (for ^{1}H) NMR instrument.

**Acquisition parameters**	**FR-ME-HSQC**
Pulse sequence	hsqcedgpphsp_rev.2
Acquisition mode (FnMODE)	States-TPPI
Time domain points (TD)	2048 (F2) &<br>1024 (F1)
Dummy scans (DS)	64
Number of scans (NS)	16;<br>32 for time-equivalent NUS spectra
Spectral width (SW), kHz	9.6 (F2) &<br>31.2 (F1)
Acquisition time (AQ), sec	0.106 (F2) &<br>0.016 (F1)
One-bond C-H coupling, $^1J_{CH}$, Hz	145
D1, sec	2
Options for zg	-DFLAG_INV
Trim pulse, µs	-
180° shaped pulse for inversion	500 µs (Crp60,0.5,20.1)
180° shaped pulse for refocusing	-
180° shaped pulse for adiabatic matched sweep‖	1498 µs (Crp60_xfilt.2)
**Processing parameters**	
Zero filling (SI)	4096 (F2) &<br>2048 (F1)
Window function (WDW)	QSINE; SSB 2 (F2) &<br>QSINE; SSB 2 (F1)
Phasing mode (PH_mod)	pk (F2) &<br>pk (F1)
Reverse spectrum during transform	False (F2) &<br>False (F1)
MC2 (FnMODE)	States-TPPI
**Specific nonuniform sampling (NUS) parameters**	
NUS schedule#	Sinusoidal-weighted Poisson-gap sampling (sPGS)
NUS reconstruction	Compressed sensing-iterative soft thresholding (CS-IST)

‖180° shaped pulse for adiabatic matched sweep: 1730 µs at 600.13 MHz, otherwise: 1730*sqrt(600/x). Where x is the magnetic field employed (800 MHz for the experiments on marine DOM).
#Schedule Generator Version 3.0 provided on nus@HMS webpage:
http://gwagner.med.harvard.edu/intranet/hmsIST/gensched_new.html

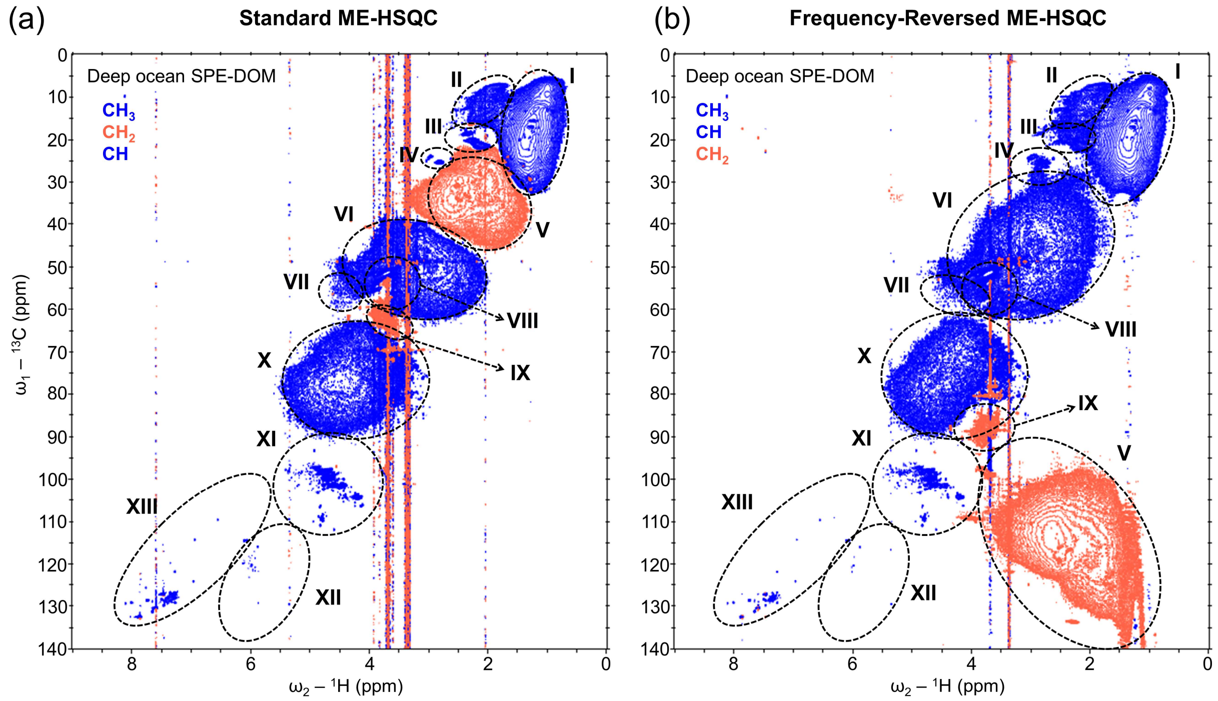

Figure S1. NMR spectra of deep ocean SPE-DOM. Comparison of the standard ME-HSQC (a) and FR-ME-HSQC (b) spectra of deep ocean SPE-DOM in CD_3OD solvent, recorded on a 900 MHz (for ^1H) NMR instrument. The blue (positive amplitude) cross-peaks represent the CH_3 and CH correlations and red (negative amplitude) cross-peaks represent the CH_2 correlations. Reversing the frequency of the CH_2 cross-peaks (red) into the signal-free region of the ME-HSQC (b) significantly simplifies the overcrowded spectral regions and eliminates signal cancellation caused by accidental overlap. Key structural assignments are as follows: **I**, diverse aliphatic groups and terminal methyl groups; **II**, N-acetyl/O-acetyl/S-**CH_3** and C=C-**CH_3**; **III**, acetates (-OOC-**CH_3**) and aromatic methyl groups (Ar-**CH_3**); **IV**, N-methyl groups (-HN-**CH_3**); **V**, diverse **CH_2** groups and carboxyl-rich alicyclic molecules (CRAM); **VI**, diverse **CH** groups and CRAM; **VII**, alpha groups (**$C_\alpha H_\alpha$**) in biomolecules; **VIII**, methyl esters (-OCO-**CH_3**) and methoxy groups (-O-**CH_3**); **IX**, **CH_2** in carbohydrates, and bonded to oxygen (-O-**CH_2**-); **X**, diverse **CH** groups, mainly from carbohydrates; **XI**, anomeric **CH** in carbohydrates; **XII**, olefinic **CH**; **XIII**, **CH** groups in aromatic, heterocyclic and polycyclic aromatic hydrocarbons (PAHs).

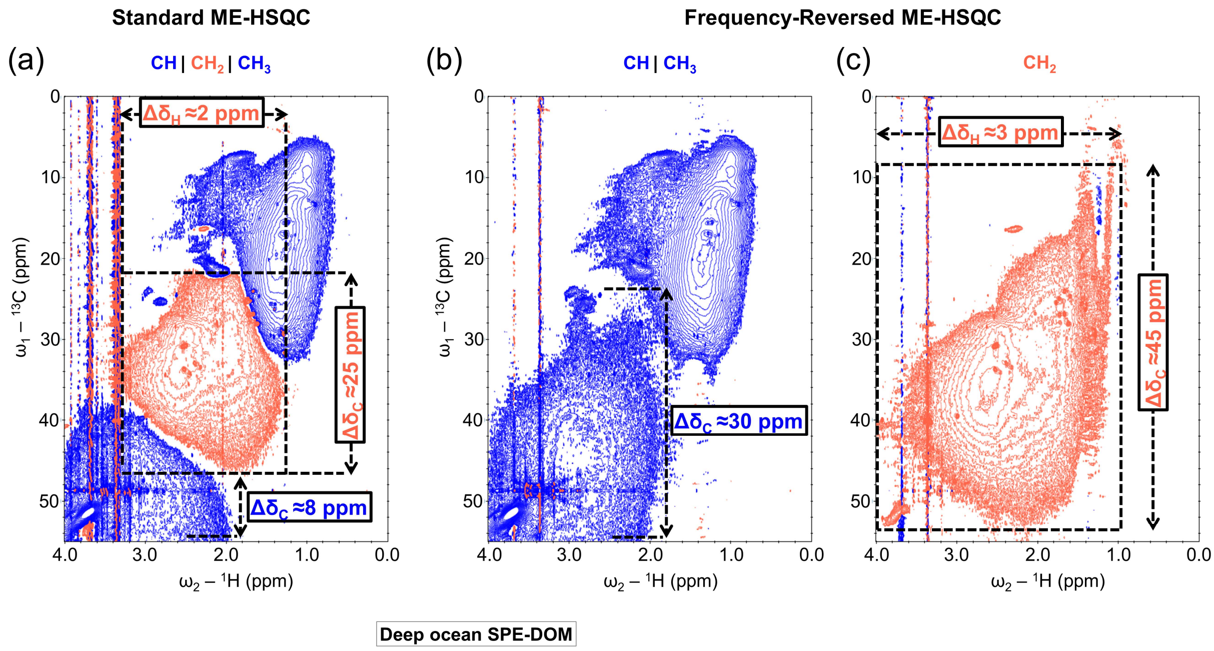

Figure S2. NMR spectra of deep ocean SPE-DOM. Comparison of the selected region of standard ME-HSQC (a), FR-ME-HSQC with CH and CH_3 correlations (b), and FR-ME-HSQC with CH_2 correlations (c) of deep ocean SPE-DOM in CD_3OD solvent, recorded on a 900 MHz (for ^1H) NMR instrument. The blue (positive amplitude) cross-peaks represent the CH_3 and CH correlations and red (negative amplitude) cross-peaks represent the CH_2 correlations. The spectrum in (c) is inverted in the indirect dimension during the Fourier transformation to display the original frequency of the CH_2 correlations. Dotted lines indicate the range of ^{13}C and ^1H chemical shifts for different carbon-proton correlations. In the standard ME-HSQC, the carbon chemical shifts spanned approximately 25 ppm, while proton chemical shifts covered about 2 ppm (a). In contrast, the FR-ME-HSQC effectively retained the diverse structural information of CH_2 groups, as indicated by the expanded range of carbon chemical shifts (approximately 45 ppm) and proton chemical shifts (around 3 ppm) (c). Furthermore, in a specific region of the standard ME-HSQC, the carbon chemical shifts of CH correlations were confined to a smaller range (approximately 8 ppm) (a), whereas the FR-ME-HSQC exhibited an increased carbon chemical shift range for these CH cross-peaks, spanning approximately 30 ppm (b). This clear enhancement highlights the potential of FR-ME-HSQC in providing comprehensive structural information for marine DOM.

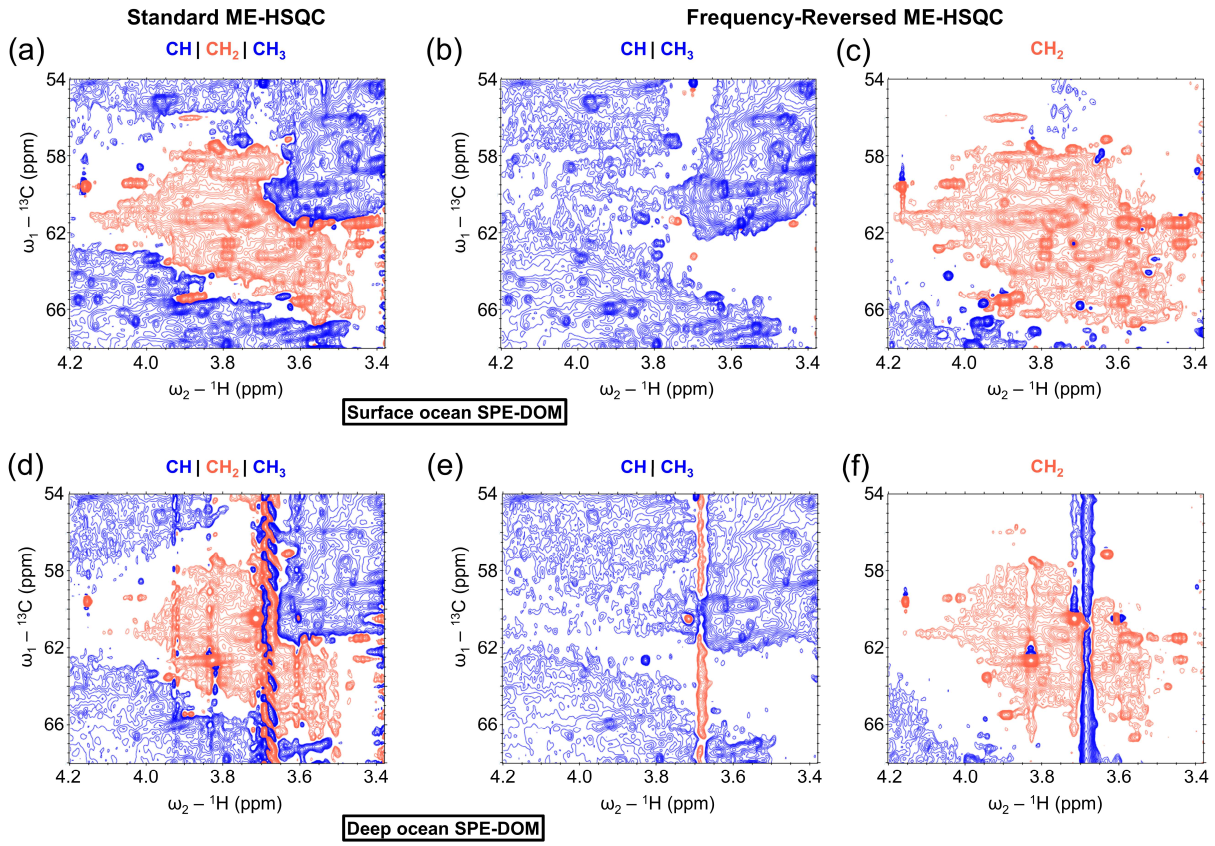

Figure S3. NMR spectra of surface (top panel) and deep (bottom panel) ocean SPE-DOM. Comparison of a selected region of standard ME-HSQC (a and d), FR-ME-HSQC with CH and CH_3 cross-peaks (b and e), and FR-ME-HSQC with CH_2 cross-peaks (c and f) of surface and deep ocean SPE-DOM, respectively, in CD_3OD solvent, recorded on a 900 MHz (for ^1H) NMR instrument. The blue (positive amplitude) cross-peaks represent the CH_3 and CH correlations and red (negative amplitude) cross-peaks represent the CH_2 correlations. The spectra in (c and f) are inverted in the indirect dimension (ω_1) during the Fourier transformation to display the original frequency of the CH_2 correlations. The frequency-reversed ME-HSQC revealed profound differences in the molecular composition between surface and deep ocean SPE-DOM, which otherwise appear structurally more similar when analyzed using the standard ME-HSQC.

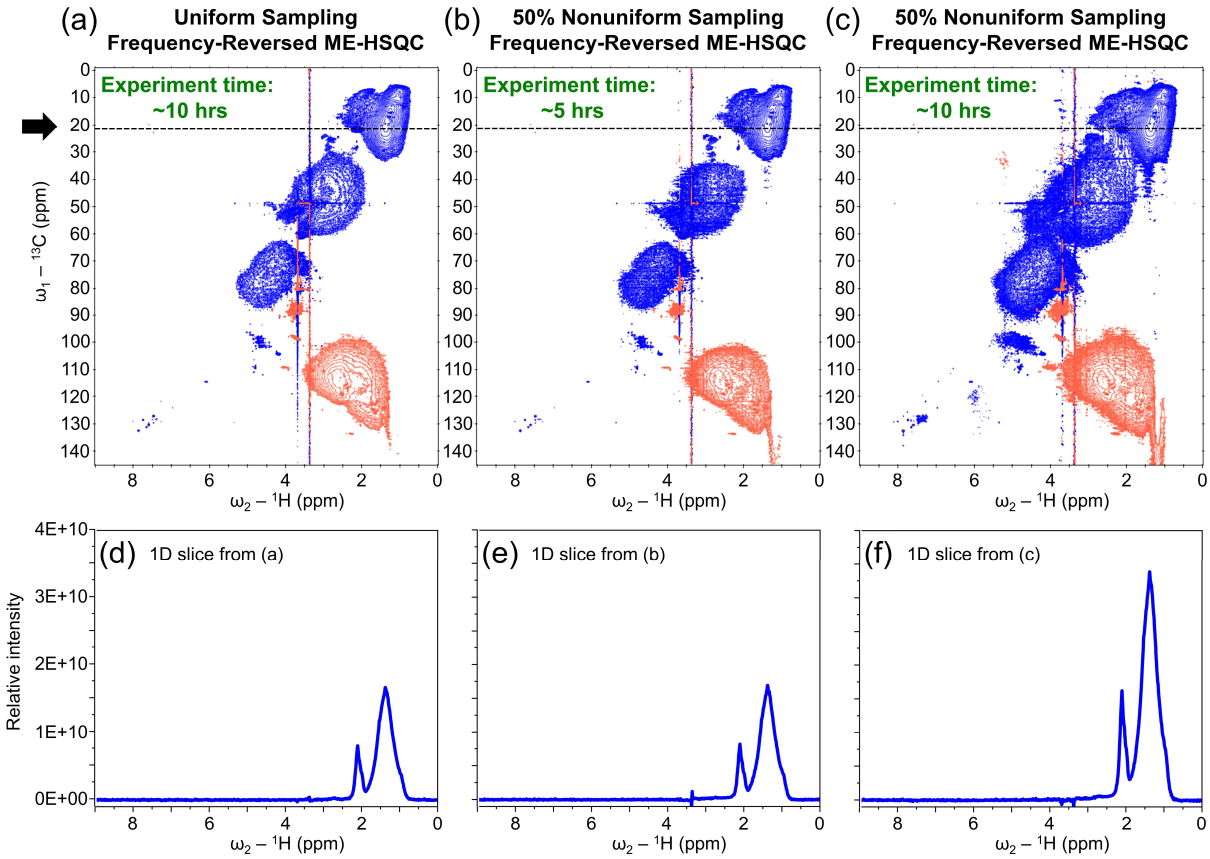

Figure S4. NMR spectra of deep ocean SPE-DOM. Comparison of the conventional uniformly sampled FR-ME-HSQC (a), 50% NUS FR-ME-HSQC recorded in half the time (b), and time-equivalent 50% NUS FR-ME-HSQC (c) of deep ocean SPE-DOM in CD_3OD solvent, recorded on an 800 MHz (for ^1H) NMR instrument. The 1D slices (d), (e), and (f) are extracted along the direct dimension (ω_2) of (a), (b), and (c), respectively, at a position indicated by the dotted lines and an arrow.

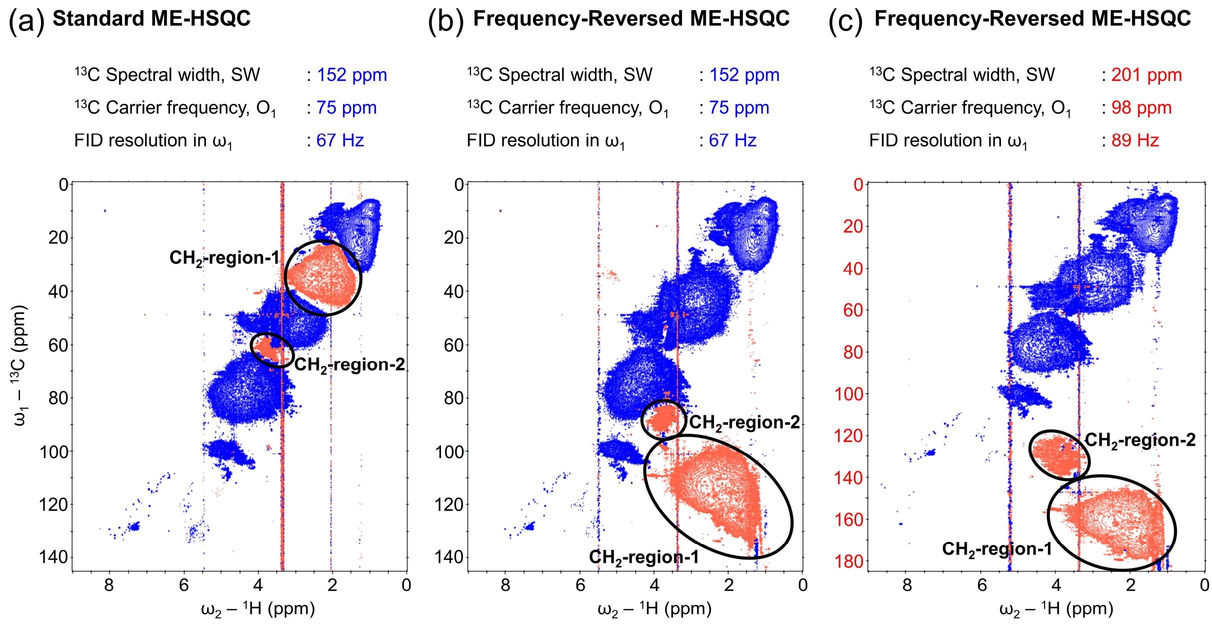

Figure S5: NMR spectra of surface Ocean SPE-DOM. The choice of ^{13}C spectral width and carrier frequency for effective separation of CH_2 regions without overlap with CH and CH_3 correlations. The key experimental parameters are indicated above the spectra. To highlight the increased spectral width in (c), the indirect dimension (ω_1) scale is depicted in red. A standard ME-HSQC (a) is included for comparison. The FR-ME-HSQC (b), acquired with a spectral width of 152 ppm and a carrier frequency of 75 ppm, enables the complete separation of CH_2-region-1. However, CH_2-region-2 remains partially overlapped with the positive CH/CH_3 correlations, resulting in a minor signal loss. As depicted in (c), adjusting the spectral width and carrier frequency of ^{13}C allows for effective separation of both CH_2 regions from CH and/or CH_3 correlations. Nonetheless, increasing the spectral width leads to a reduction in fid resolution or an extended experiment time to achieve the desired fid resolution in the indirect dimension.

Table S3. Plausible molecular structures in which the carbon-proton cross-peaks of **CH_3** groups (blue) can fall into those of **CH_2** groups are prone to signal cancellation in the standard ME-HSQC of marine DOM. Carbon and proton chemical shifts are obtained from the Biological Magnetic Resonance Data Bank (BMRB)[8-14].

**Name of the compound**	**Chemical structure**	**δ_C, ppm**	**δ_H, ppm**
Vanillic acid	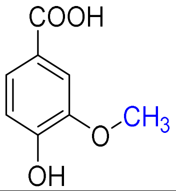	58.6	3.90
3,4,5-Trimethoxycinnamic acid	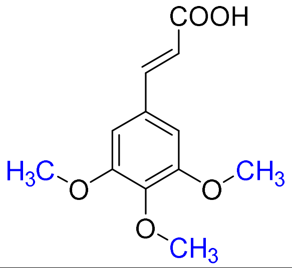	56.2<br>56.2<br>61.0	3.89<br>3.89<br>3.89
Pyruvic acid	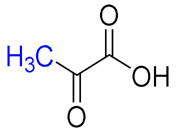	29.2	2.36
Sarcosine	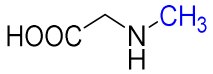	35.4	2.73
N-Acetylglucosamine	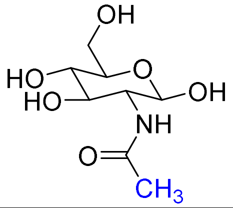	24.9	2.04
Creatinine	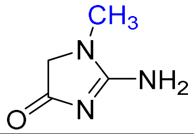	32.9	3.03
Citraconic acid	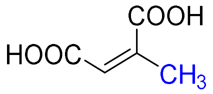	23.1	1.91
Creatine	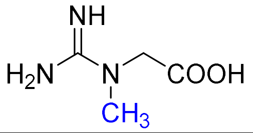	39.7	3.02

Table S4. Plausible molecular structures in which the carbon-proton cross-peaks of **CH_2** groups (red) can fall into those of **CH_3** and **CH** groups are prone to signal cancellation in the standard ME-HSQC of marine DOM. Carbon and proton chemical shifts are obtained from the Biological Magnetic Resonance Data Bank (BMRB).

**Name of the compound**	**Chemical structure**	**δ_C, ppm**	**δ_H, ppm**
Phenylacetic acid	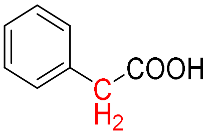	47.2	3.52
4-Hydroxyphenylacetic acid	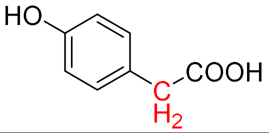	46.2	3.44
L-Phenylalanine	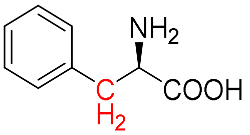	39.1	3.27<br>3.11
Hippuric acid	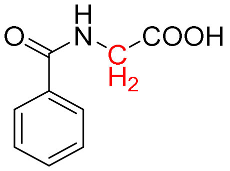	46.5	3.94
L-Tyrosine	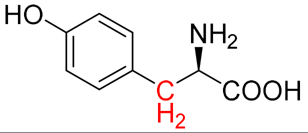	38.3	3.20<br>3.06
Tyramine	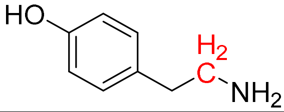	43.5	3.23
Dopamine	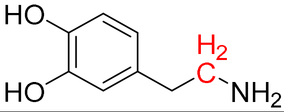	40.2	2.90
Tryptamine	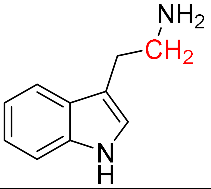	42.4	3.33
Serotonin	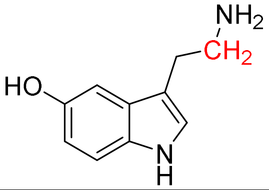	42.3	3.31
Guanidoacetic acid	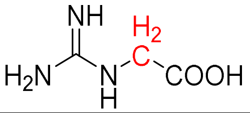	47.3	3.78
L-Arginine	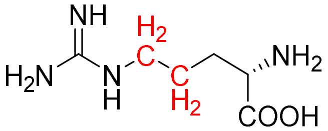	43.2<br>26.6	3.24<br>1.68

Glycine	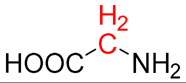	44.1	3.55
L-Lysine	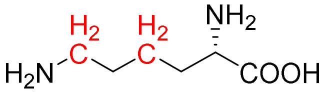	41.8<br>24.1	3.01<br>1.47
L-Isoleucine	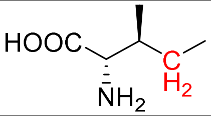	27.2	1.46<br>1.25
O-Phosphoethanolamine	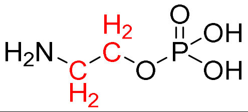	62.9<br>43.3	3.97<br>3.21
Phosphonoacetic acid	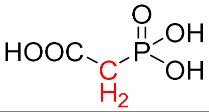	42.6	2.66<br>2.62
L-Cystine	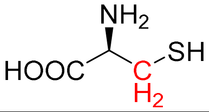	27.6	3.04
L-(+)-Arabinose	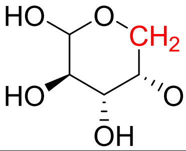	69.1	3.83<br>3.78
Citric acid	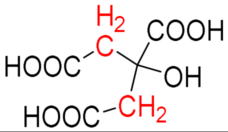	48.4	2.58
L-Ascorbic acid	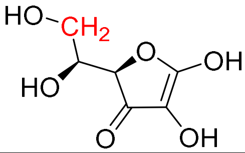	65.3	3.74
Diethanolamine	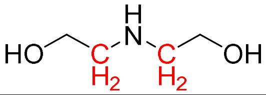	51.6	3.23
DL-Pipecolic acid	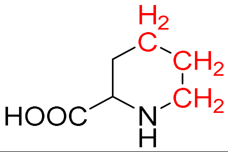	46.4<br>24.5<br>24.3	3.20<br>1.73<br>1.76
Dihydrouracil	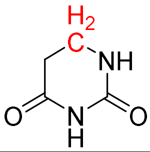	38.2	3.45
L-(-) Arabitol	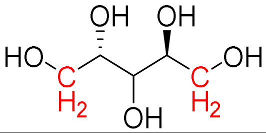	65.8<br>65.7	3.83<br>3.66
Glutaconic acid	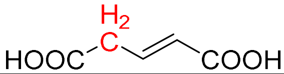	43.6	3.10<br>3.07
Trans-3-hexenedioic acid	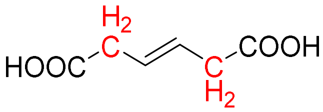	44.1	2.94

L-Proline	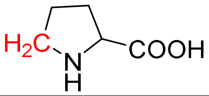	48.8	3.37
Histamine	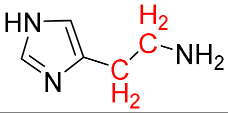	41.7<br>26.9	3.28<br>3.01
Dodecanoic acid	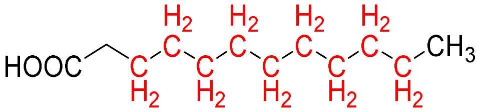	22.7<br>31.9<br>29.2<br>29.4<br>31.9<br>29.6<br>29.3<br>29.0<br>24.7	1.26<br>1.26<br>1.26<br>1.26<br>1.26<br>1.26<br>1.26<br>1.26<br>1.63
Isovaleric acid	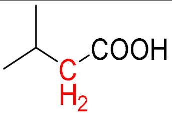	49.9	2.04
Cholic acid	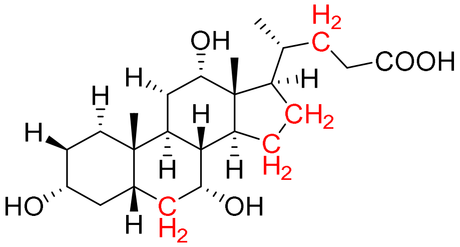	35.1<br><br>32.2<br><br><br>27.3<br><br><br>23.0	0.984<br><br>1.50<br>1.38<br><br>1.50<br>1.38<br><br>1.07
Malonic acid	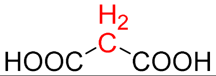	50.2	3.11
3-Methylglutaric acid	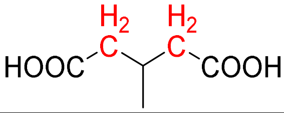	48.0	2.22<br>1.98
Taurine	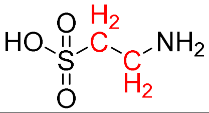	50.2<br><br>38.1	3.25<br><br>3.41

Table S5. Plausible molecular structures in which the carbon-proton cross-peaks of **CH** groups (blue) can fall into those of **CH_2** groups are prone to signal cancellation in the standard ME-HSQC of marine DOM. Carbon and proton chemical shifts are obtained from the Biological Magnetic Resonance Data Bank (BMRB).

**Name of the compound**	**Chemical structure**	**δ_C, ppm**	**δ_H, ppm**
2-hydroxy-3-methylbutyric acid	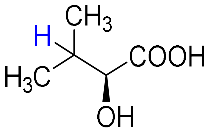	34.1	2.00
L-Valine	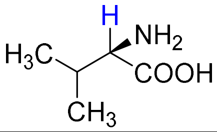	63.0	3.60
L-Threonine	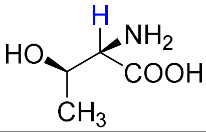	63.2	3.57
L-Leucine	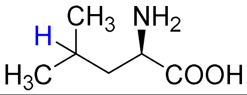	26.9	1.70
L-Isoleucine	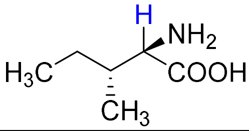	61.6	4.16
2-methylglutaric acid	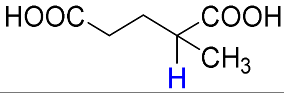	45.5	2.25
L-Tyrosine	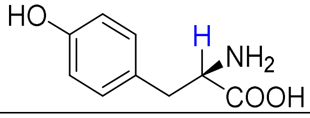	58.8	3.94
Isovaleric acid	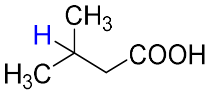	28.9	1.94
O-phosphoserine	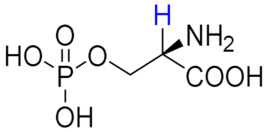	58.4	3.93

The following is the Bruker pulse program used for recording the FR-ME-HSQC spectra in this study.

```
;hsqcedgpphsp_rev.2
;avance-version (20/08/05)
;HSQC
;2D H-1/X correlation via double inept transfer
;phase sensitive using Echo/Antiecho-TPPI gradient selection
;with decoupling during acquisition
;using trim pulses in inept transfer
;with multiplicity editing during selection step
;using shaped pulses for inversion on f2 - channel
;for matched sweep adiabatic pulses
;reversing chemical shift for CH2 groups
;
;P. Sakhaii & W. Bermel, J. Magn. Reson. 259, 82-86 (2015)
;(C. Zwahlen, P. Legault, S.J.F. Vincent, J. Greenblatt, R. Konrat &
;   L.E. Kay, J. Am. Chem. Soc. 119 6711-6721 (1997) )
;
;$CLASS=HighRes
;$DIM=2D
;$TYPE=
;$SUBTYPE=
;$COMMENT=

#include <Avance.incl>
#include <Grad.incl>
#include <Delay.incl>

"p2=p1*2"
"d2=1s/(cnst20*2)"
"d4=1s/(cnst2*4)"
"d11=30m"

"d0=3u"

"in0=inf1/2"

"DELTA1=d4-larger(p2,p14)/2"
"DELTA2=d2/2+p31/2"
"DELTA3=d2/2-p31/2-p19-d16"
"DELTA4=d2/2+p31/2-p19-d16"
"DELTA5=d2/2-p31/2"
"DELTA6=d2-p19-d16"

"TAU1=d0+p3*2/PI+1u"
"TAU2=d0+p2+p1*2+p3*2/PI+1u"

"spoff18=bf2*(75.0/1000000)-o2"
```

```
#   ifdef FLAG_INV
"l0=1"
#   else
"l0=0"
#   endif /*FLAG_INV*/

"acqt0=-p1*2/PI"

1 ze
  d11 pl12:f2
2 d1 do:f2
  50u UNBLKGRAD

  (p1 ph1)
  DELTA1
  (center (p2 ph1) (p14:sp3 ph6):f2 )
  DELTA1

#   ifdef TRIMP
  p28 ph1
#   endif /* TRIMP */

  (p1 ph2)

  p16:gp3
  d16 pl2:f2

  (p3 ph3):f2
  d0
  (p2 ph5)
  d0

  if "l0 %2 == 0"
     {
     (p1 ph1):f1
     DELTA2
     (p1 ph1 p2 ph2 p1 ph1):f1
     p19:gp6
     d16
     DELTA3
     (p31:sp18 ph1):f2
     TAU1
     (p1 ph2 p2 ph1 p1 ph2):f1
     TAU1
     DELTA4
     p19:gp6
     d16
     (p1 ph1 p2 ph2 p1 ph1):f1
     DELTA5
     (p31:sp18 ph1):f2
     (p1 ph1):f1
```

```
    }
  else
    {
    (p1 ph1):f1
    (p1 ph1 p2 ph2 p1 ph1):f1
    p19:gp6
    d16
    DELTA6
    (p31:sp18 ph1):f2
    TAU2
    (p1 ph2 p2 ph1 p1 ph2):f1
    TAU2
    DELTA6
    p19:gp6
    d16
    (p31:sp18 ph1):f2
    (p1 ph1 p2 ph2 p1 ph1):f1
    (p1 ph1):f1
    }

  2u pl2:f2
  (p3 ph4):f2

  p16:gp4
  d16

  (p1 ph1)
  DELTA1
  (center (p2 ph1) (p14:sp3 ph1):f2 )
  DELTA1
  (p1 ph2)

  p16:gp5
  d16 pl12:f2
  4u BLKGRAD

  (p1 ph1)
  go=2 ph31 cpd2:f2
  d1 do:f2 mc #0 to 2
    F1PH(calph(ph3, +90) & calclc(l0, 1), caldel(d0, +in0))
exit

ph1=0
ph2=1
ph3=0 2
ph4=0 0 0 0 2 2 2 2
ph5=0 0 2 2
ph6=0
ph31=2 0 2 0 0 2 0 2
```

```
;pl1 : f1 channel - power level for pulse (default)
;pl2 : f2 channel - power level for pulse (default)
;pl12: f2 channel - power level for CPD/BB decoupling
;sp3: f2 channel - shaped pulse 180 degree
;spnam3: Crp60,0.5,20.1 (Crp80,0.5,20.1)
;sp18: f2 channel - shaped pulse 180 degree (adiabatic matched sweep)
;spnam18: Crp60_xfilt.2
;p1 : f1 channel -  90 degree high power pulse
;p2 : f1 channel - 180 degree high power pulse
;p3 : f2 channel -  90 degree high power pulse
;p14: f2 channel - 180 degree shaped pulse for inversion
;p16: homospoil/gradient pulse
;p19: gradient pulse 2                        [500 usec]
;p28: f1 channel - trim pulse
;p31: f2 channel - 180 degree shaped pulse for adiabatic matched sweep
;              1730 us at 600.13 MHz, otherwise: 1730*sqrt(600/x)
;d0 : incremented delay (2D)              [3 usec]
;d1 : relaxation delay; 1-5 * T1
;d2 : 1/(2J)CH
;d4 : 1/(4J)CH
;d11: delay for disk I/O                      [30 msec]
;d16: delay for homospoil/gradient recovery
;cnst2: = J(CH)                           [145 Hz]
;cnst17: = -0.5 for Crp60comp.4
;cnst20: = J'(CH)                         [147.4 Hz]
;inf1: 1/SW(C) = 2 * DW(C)
;in0: 1/(2 * SW(C)) = DW(C)
;nd0: 2
;ns: 2 * n
;ds: >= 16
;td1: number of experiments
;FnMODE: States-TPPI (or TPPI)
;cpd2: decoupling according to sequence defined by cpdprg2
;pcpd2: f2 channel - 90 degree pulse for decoupling sequence

;for z-only gradients:
;gpz3: 60%
;gpz4: 40%
;gpz5: 31%
;gpz6: 19%

;use gradient files:
;gpnam3: SMSQ10.100
;gpnam4: SMSQ10.100
;gpnam5: SMSQ10.100
;gpnam6: SMSQ10.100

;cnst17: Factor to compensate for coupling evolution during a pulse
;       (usually +1). A positive factor indicates that coupling
;       evolution continues during the pulse, whereas a negative
;       factor is necessary if the coupling is (partially) refocussed.
```

```
                                    ;preprocessor-flags-start
;LABEL_CN: for C-13 and N-15 labeled samples start experiment with
;            option -DLABEL_CN (eda: ZGOPTNS)
;FLAG_INV: invert signal amplitude as well as frequency for CH2 goups
;            option -DFLAG_INV (eda: ZGOPTNS)
                                    ;preprocessor-flags-end

;for matched sweep use
;   low to high field sweep,
;   carrier shifted to center of spectrum (75 ppm) via spoffs18

;$Id: hsqcedgpphsp_rev.2,v 1.2 2020/08/05 14:49:23 ber Exp $
```

REFERENCES

(1) Palmer, A. G.; Cavanagh, J.; Wright, P. E.; Rance, M. *J. Magn. Reson. (1969)* **1991**, *93* (1), 151-170.

(2) Kay, L. E.; Keifer, P.; Saarinen, T. *J. Am. Chem. Soc.* **1992**, *114* (26), 10663-10665.

(3) Schleucher, J.; Schwendinger, M.; Sattler, M.; Schmidt, P.; Schedletzky, O.; Glaser, S. J.; Sørensen, O. W.; Griesinger, C. *J. Biomol. NMR* **1994**, *4* (2), 301-306.

(4) Willker, W.; Leibfritz, D.; Kerssebaum, R.; Bermel, W. *Magn. Reson. Chem.* **1993**, *31* (3), 287-292.

(5) Zwahlen, C.; Legault, P.; Vincent, S. J. F.; Greenblatt, J.; Konrat, R.; Kay, L. E. *J. Am. Chem. Soc.* **1997**, *119* (29), 6711-6721.

(6) Boyer, R. D.; Johnson, R.; Krishnamurthy, K. *J. Magn. Reson.* **2003**, *165* (2), 253-9.

(7) Sakhaii, P.; Bermel, W. *J. Magn. Reson.* **2015**, *259*, 82-6.

(8) Hoch, J. C.; Baskaran, K.; Burr, H.; Chin, J.; Eghbalnia, Hamid R.; Fujiwara, T.; Gryk, Michael R.; Iwata, T.; Kojima, C.; Kurisu, G.; Maziuk, D.; Miyanoiri, Y.; Wedell, Jonathan R.; Wilburn, C.; Yao, H.; Yokochi, M. *Nucl. Acids Res.* **2023**, *51*, D368-D376.

(9) Baskaran, K.; Craft, D. L.; Eghbalnia, H. R.; Gryk, M. R.; Hoch, J. C.; Maciejewski, M. W.; Schuyler, A. D.; Wedell, J. R.; Wilburn, C. W. *Front. Mol. Biosci.* **2022**, *8*.

(10) Romero, P. R.; Kobayashi, N.; Wedell, J. R.; Baskaran, K.; Iwata, T.; Yokochi, M.; Maziuk, D.; Yao, H.; Fujiwara, T.; Kurusu, G.; Ulrich, E. L.; Hoch, J. C.; Markley, J. L. In *Structural Bioinformatics: Methods and Protocols*, Gáspári, Z., Ed. Springer US: New York, NY, **2020**, 187-218.

(11) Ulrich, E. L.; Baskaran, K.; Dashti, H.; Ioannidis, Y. E.; Livny, M.; Romero, P. R.; Maziuk, D.; Wedell, J. R.; Yao, H.; Eghbalnia, H. R.; Hoch, J. C.; Markley, J. L. *J. Biomol. NMR* **2019**, *73* (1), 5-9.

(12) Velankar, S.; Burley, S. K.; Kurisu, G.; Hoch, J. C.; Markley, J. L. In *Structural Proteomics: High-Throughput Methods*, Owens, R. J., Ed. Springer US: New York, NY, **2021**, 3-21.

(13) Ulrich, E. L.; Akutsu, H.; Doreleijers, J. F.; Harano, Y.; Ioannidis, Y. E.; Lin, J.; Livny, M.; Mading, S.; Maziuk, D.; Miller, Z.; Nakatani, E.; Schulte, C. F.; Tolmie, D. E.; Kent Wenger, R.; Yao, H.; Markley, J. L. *Nucl. Acids Res.* **2008**, *36*, D402-D408.

(14) wwPDB consortium, *Nucl. Acids Res.* **2019**, *47*, D520-D528.
